# Association of Administration of Surfactant Using Less Invasive Methods With Outcomes in Extremely Preterm Infants Less Than 27 Weeks of Gestation

**DOI:** 10.1001/jamanetworkopen.2022.25810

**Published:** 2022-08-09

**Authors:** Christoph Härtel, Egbert Herting, Alexander Humberg, Kathrin Hanke, Katrin Mehler, Titus Keller, Isabell Mauer, Eric Frieauff, Sascha Meyer, Ulrich H. Thome, Christian Wieg, Susanne Schmidtke, Angela Kribs, Wolfgang Göpel

**Affiliations:** 1 Department of Paediatrics, University of Würzburg, Würzburg, Germany; 2 Department of Paediatrics, University Hospital Schleswig-Holstein/Campus Lübeck, Lübeck, Germany; 3 Division of Neonatology, Department of Paediatrics, University of Cologne, Cologne, Germany; 4 Department of General Pediatrics and Neonatology, University Children’s Hospital of Saarland, Homburg, Germany; 5 Division of Neonatology, Department of Women’s and Children’s Health, University Children’s Hospital Leipzig, Leipzig, Germany; 6 Neonatology and Pediatric Intensive Care, Children’s Hospital Aschaffenburg-Alzenau, Aschaffenburg, Germany; 7 Division of Neonatology, Asklepios Hospital Hamburg-Barmbek, Hamburg, Germany

## Abstract

**Importance:**

The inclusion of less invasive surfactant administration (LISA) in the care of preterm infants has been found to be beneficial for respiratory outcomes. Recently, the OPTIMIST trial found higher mortality rates in the subgroup of infants born at 25 to 26 weeks’ gestational age (GA) who received surfactant treatment while spontaneously breathing.

**Objective:**

To analyze outcomes among LISA-exposed, highly vulnerable babies born at less than 27 weeks’ GA within the large-scale observational cohort of the German Neonatal Network.

**Design, Setting, and Participants:**

In this cohort study of data from 68 tertiary level neonatal intensive care units in Germany of infants born between 22 weeks 0 days to 26 weeks 6 days of gestation between April 1, 2009, and December 31, 2020, short-term outcomes among infants receiving LISA vs infants not receiving LISA were compared.

**Exposure:**

Use of LISA within the first 72 hours of life.

**Main Outcomes and Measures:**

The main outcomes were rates of LISA use, use of mechanical ventilation within the first 72 hours (considered failure of LISA), and association of LISA with outcomes, including death from all causes, bronchopulmonary dysplasia (BPD), death and BPD combined, pneumothorax, retinopathy of prematurity, intracerebral hemorrhage, and periventricular leukomalacia. To address potential confounding factors, multivariate logistic regression models were used.

**Results:**

A total of 6542 infants (3030 [46.3%] female and 3512 [53.7%] male; mean [SD] GA, 25.3 (1.1) weeks; mean [SD] birth weight, 715 [180] g) were analyzed; 2534 infants (38.7%) received LISA, which was most frequently given quasi-prophylactically during delivery room management. Among the infants who received LISA, 1357 (53.6%) did not require mechanical ventilation in the first 72 hours compared with 331 infants (8.3%) of 4008 who did not receive LISA. In a multivariate logistic regression model that adjusted for GA, small-for-GA status, sex, multiple birth, inborn status, antenatal steroid use, and maximum fraction of inspired oxygen in the first 12 hours of life, LISA was associated with reduced risks of all-cause death (odds ratio [OR], 0.74; 95% CI, 0.61-0.90; *P* = .002), BPD (OR, 0.69; 95% CI, 0.62-0.78; *P* < .001), and BPD or death (OR, 0.64; 95% CI, 0.57-0.72; *P* < .001) compared with infants without LISA exposure.

**Conclusions and Relevance:**

The results of this long-term multicenter cohort study suggest that LISA may be associated with reduced risks of adverse outcomes in extremely preterm infants.

## Key Points

**Question:**

Is the use of less invasive surfactant administration (LISA) in extremely preterm infants of less than 27 weeks’ gestational age associated with adverse outcomes of prematurity?

**Findings:**

In this cohort study of 6542 infants born between 22 weeks 0 days and 26 weeks 6 days at 68 German tertiary level neonatal intensive care units, 2534 infants received LISA. Use of LISA was associated with reduced risk of all-cause death, bronchopulmonary dysplasia, and the combined outcome bronchopulmonary dysplasia or death.

**Meaning:**

The findings of this study suggest that, in experienced centers, the use of LISA is associated with increased survival in extremely preterm infants.

## Teaser

This cohort study of infants 22 weeks to 26 weeks 6 days’ gestational age from the German Neonatal Network compares outcomes between those who did and did not receive the study intervention LISA (less invasive surfactant administration).

## Introduction

The beneficial treatment effects of less invasive thin catheter surfactant administration techniques such as less invasive surfactant administration (LISA) or minimally invasive surfactant treatment (MIST) for preterm infants with respiratory distress syndrome have been demonstrated in several randomized clinical trials RCTs)^1,2,3,4^ and further supported in meta-analyses.^5,6^ Less invasive surfactant administration is not a single technical procedure but rather a bundle concept comprising less invasive care that emphasizes delayed cord clamping, facilitated fetal transition, initial continuous positive airway pressure (CPAP) support, maintenance of spontaneous breathing, caffeine administration, and early skin-to-skin contact.^7,8^ Use of LISA significantly reduces the exposure to invasive mechanical ventilation (IMV) in the first 72 hours in infants older than 25 weeks’ gestational age (GA) and, in particular, those older than 27 weeks’ GA.^1,3,4^ Even in infants 25 weeks’ GA or younger, IMV can be avoided in 40% of cases.^3,9,10^ It is currently debated whether the benefits of LISA and MIST wane with decreasing GA.^4^ The large, international OPTIMIST trial (Collaborative Paired Trials Investigating Minimally Invasive Surfactant Therapy)^4^ randomized infants born at 25 weeks 0 days to 28 weeks 6 days’ GA receiving CPAP therapy and a fraction of inspired oxygen (Fio_2_) level of 0.3 or greater to continued noninvasive ventilatory support or selective treatment with MIST. The investigators found reduced incidences of bronchopulmonary dysplasia (BPD) and the composite BPD or death in infants treated with MIST. Notably, all-cause hospital mortality was higher in the youngest infants enrolled in OPTIMIST, those born at 25 to 26 weeks, when exposed to MIST compared with control patients (17.8% vs 8.0%; odds ratio [OR], 2.18 [95% CI, 0.97-4.88]). Although these differences were not statistically significant, they would be clinically relevant for extremely preterm infants.^11^ This is in contrast to findings of the Nonintubated Surfactant Application (NINSAPP) trial of spontaneously breathing infants born at 23 to 26 weeks’ GA, which found no adverse effect of LISA on mortality, while the risk of any severe complication was reduced.^3^

However, there is still uncertainty about the optimal treatment criteria, timing, and delivery mode of surfactant in the tiniest babies. As tailored approaches to LISA are urgently needed, a better risk stratification and safety profile derived from large-scale, population-based data is essential. We therefore analyzed the short-term outcome data from 2534 LISA-exposed infants of 22 to 26 weeks’ GA within the German Neonatal Network (GNN) collaborative effort, including more than 10 years’ experience with the LISA strategy.

## Methods

### Patient Population

**Figure 1.**
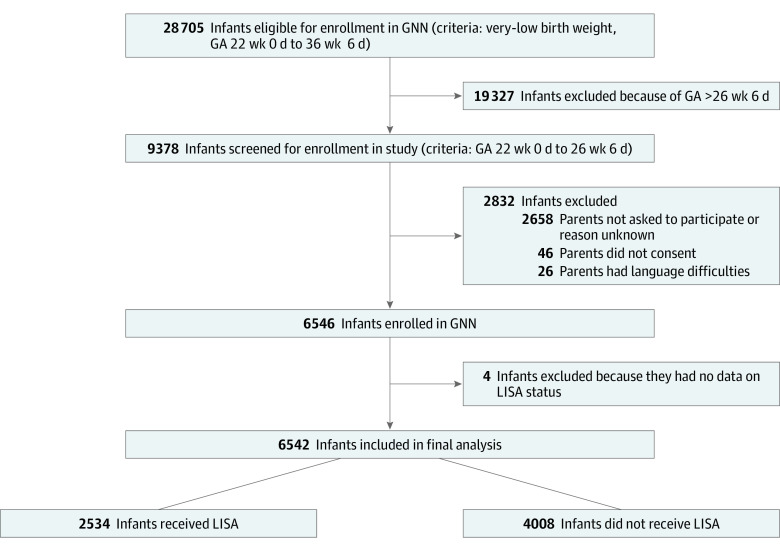
Study Flow Diagram GA indicates gestational age; GNN, German Neonatal Network; LISA, less invasive surfactant administration.

The GNN is a multicenter collaboration of 68 tertiary level neonatal care units across Germany to study risks and complications of very low-birth-weight infants born at 22 weeks 0 days to 36 weeks 6 days of gestation at a population-based level. Main short-term outcomes are assessed at discharge from primary stay in hospital. A subgroup of infants has been followed until early school age. For the purposes of this cohort study, we analyzed short-term outcome data from infants with a gestational age of 22 weeks 0 days to 26 weeks 6 days born in GNN centers between April 1, 2009, and December 31, 2020 (Figure 1). All study parts were approved by the University of Lübeck Ethics Committee and the committees of participating centers. This study followed the Strengthening the Reporting of Observational Studies in Epidemiology (STROBE) reporting guideline on cohort studies.

After written informed parental consent was obtained, infants were enrolled in the GNN, and data from 250 predefined parameters were recorded for each patient in clinical record files. After discharge, data sheets were sent to the leading study site (University of Lübeck, Lübeck, Germany). Data quality was evaluated by a study team neonatologist (C.H. or A.H.) via annual on-site monitoring. In this context, a baseline data set of eligible but nonenrolled infants was documented. These data were used in our analyses to address potential selection bias (eTables 1 and 2 in Supplement 1).

### Study Definitions

Small-for-gestational-age (SGA) status was defined as a birth weight less than the 10th percentile according to sex-specific standards for birth weight by postmenstrual age in Germany. Intracerebral hemorrhage (ICH) was defined as ultrasound diagnosis of ICH according to Papile grading (grades I to IV, with I being the least and IV being the most severe). Periventricular leukomalacia was defined as periventricular, cystic white matter lesion diagnosed by cranial ultrasound. Bronchopulmonary dysplasia was defined as the need for oxygen or respiratory support (CPAP or mechanical ventilation) at 36 weeks’ postmenstrual GA. Mortality was defined as deaths from all causes during the primary stay in hospital. The failure of LISA (LISA failure) was defined as the need for IMV in the first 72 hours of life after the initial application of LISA.

### Statistical Analysis

Hypotheses were evaluated in univariate analyses using 2-sided χ^2^ tests for categorical variables and the Mann-Whitney *U* test for continuous variables for comparisons. We conducted multivariate logistic regression models reporting ORs and 95% CIs. These models included known or probable confounders as independent variables, including infant characteristics and maternal factors. A 2-tailed *P* < .05 was considered statistically significant. We used a uniform data set with available data for all metric parameters. Missing data were not imputed. Data analyses were performed using SPSS software, version 27.0 (IBM Corp).

## Results

### Clinical Characteristics of the Study Cohort

A total of 9378 infants with a gestational age of 22 to 26 weeks were eligible for our study; 2832 infants were not enrolled in the GNN, the main reason being that parents were not asked for participation by site investigators (n = 2658; Figure 1). Nonenrolled infants had a lower GA (mean [SD] GA, 24.9 [1.3] vs 25.3 [1.1] weeks) and a lower birth weight (mean [SD] weight, 680 [250] vs 718 [180] g) than enrolled infants. In line with that, marked differences in short-term outcomes were observed, in particular a higher risk of all-cause death (50.9% (49.1%-53.0%) vs 9.4% (8.8%-10.1%]) in nonenrolled infants, which was noted for all subgroups of each gestational week (eTables 1 and 2 in Supplement 1). Among the whole group of eligible infants, all-cause mortality by week of GA was as follows: 22 weeks: 63.6% (95% CI, 58.3%-68.7%); 23 weeks: 42.6% (95% CI, 39.8%-45.4%); 24 weeks: 25.0% (95% CI, 23.3%-26.8%); 25 weeks: 17.5 (95% CI, 16.0%-19.0%); and 26 weeks: 9.7% (95% CI, 8.7%-10.8%).

The final study cohort for the evaluation of LISA use consisted of 6542 infants (3030 [46.3%] female and 3512 [53.7%] male; mean [SD] GA, 25.3 (1.1) weeks; mean [SD] birth weight, 715 [180] g) (Table 1). Maternal background was German in 4399 infants (68.1%); other European (including Russia) in 828 infants (11.8%), Middle East or Turkey in 715 infants (11.1%), Asian in 164 infants (2.5%), African in 284 infants (4.4%), and other background in 70 infants (1.1%). Among 4008 infants (61.3%) who did not receive LISA, 476 infants had never been treated with surfactant and 3532 infants received surfactant via endotracheal tube; 2534 (38.7%) infants received LISA. Among the 2534 infants receiving LISA, 2018 (79.6%) were cesarean births, and 2380 (93.9%) received antenatal steroids.

**Table 1.** Clinical Characteristics of Infants According to LISA Exposure (N = 6542)^a^

Characteristic	Infants, No. (%)			*P* value, LISA vs no LISA
	LISA (n = 2534)	No LISA		
		No surfactant (n = 476)	ETT surfactant (n = 3532)	
Gestational age, mean (SD), wk	25.3 (1.1)	25.6 (1.0)	25.1 (1.1)	<.001
Sex				
Female	1164 (45.9)	247 (51.9)	1619 (45.8)	.69
Male	1370 (54.1)	229 (48.1)	1913 (54.2)	
Birth weight, mean (SD), g	733 (179)	784 (165)	698 (180)	<.001
Apgar score at 5 min, mean (SD)	7 (1)	8 (2)	7 (2)	<.001
Arterial cord blood pH, mean (SD)	7.35 (0.14)	7.33 (0.1)	7.31 (0.1)	.20
Inborn status	2491 (98.3)	442 (96.3)	3225 (95.0)	<.001
Multiple birth	818 (32.3)	108 (22.7)	1060 (30.0)	.007
SGA status	399 (15.7)	171 (36.0)	677 (19.2)	.03
Antenatal steroid use	2380 (93.9)	441 (92.6)	3122 (88.4)	<.001
Mode of delivery				
Elective cesarean	2018 (79.6)	329 (69.1)	2497 (71.0)	<.001
Vaginal	240 (9.5)	99 (20.8)	505 (14.4)	
Emergency cesarean	272 (10.8)	48 (10.1)	515 (14.6)	
Cause of preterm birth^b^				
Preterm labor	1153 (45.6)	248 (52.2)	1742 (49.5)	<.001
Amniotic infection	868 (34.3)	222 (46.9)	1174 (33.3)	.60
Preeclampsia/HELLP	310 (12.2)	22 (4.6)	348 (9.9)	<.001
Pathological Doppler result	359 (14.2)	27 (5.7)	451 (12.8)	.02
Placental abruption	214 (8.5)	35 (7.4)	325 (9.2)	.60
Inotropes given in first 24 h	338 (13.5)	23 (4.9)	743 (21.5)	<.001
Mechanical ventilation, No. (%) [95% CI]				
<72 h Of life	1177 (46.5) [44.5-48.4]	145 (30.5) [26.8-34.6]	3532 (100.0)	<.001
<7 d Of life	1340 (52.9) [51.0-54.8]	163 (34.2) [30.1-38.4]	3532 (100.0)	<.001
Maximum Fio_2_ in the first 12 h of life, No. (%) [95% CI]				
Total No.^c^	2504	461	3464	
0.21-0.39	1109 (44.3) [42.4-46.2]	358 (77.7) [74.2-80.9]	1426 (41.2) [39-44]	<.001
0.4-0.59	759 (30.3) [28.5-32.1]	69 (15.0) [11.9-18.0]	847 (24.5) [23-26]	
≥0.6	636 (25.4) [23.7-27.1]	34 (7.4) [5.1-9.8]	1191 (34.4) [32-37]	

Abbreviations: ETT, endotracheal tube; Fio_2_, fraction of inspired oxygen; HELLP, highly elevated liver enzymes and low platelets; LISA, less invasive surfactant administration; SGA, small-for-gestational-age.

^a^ Continuous variables were analyzed using the Mann-Whitney *U* test; dichotomous variables were analyzed using a 2-sided χ^2^ test. *P* values were derived for comparisons between the 2534 infants who received LISA and the 4008 infants not receiving LISA (the combination of those receiving no surfactant and those receiving surfactant through ETT).

^b^ Multiple answers possible.

^c^ Based on available data set from each group.

Many GNN units have adopted a quasi-prophylactic strategy of CPAP-assisted spontaneous breathing and use of LISA to treat extremely preterm infants during delivery room management (ie, most infants received LISA without a set threshold for use, such as Fio_2_ level).^3,9^ In our study, delivery room LISA was performed in 83.3% of infants who received LISA, including 25 infants (89.3%) born at 22 weeks’ GA, 81 infants (82.7%) born at 23 weeks’ GA, 256 infants (87.1%) born at 24 weeks’ GA, 335 infants (83.3%) born at 25 weeks’ GA, and 454 infants (81.2)% born at 26 weeks’ GA. We observed that of those infants who received LISA, 1357 (53.6%) did not require IMV in the first 72 hours, whereas only 331 infants (8.3%) of the 4008 who did not receive LISA did not require IMV in the first 72 hours. This quasi-prophylactic use of LISA remained the only surfactant dose in 1523 LISA-exposed infants (60.1%). A second dose of surfactant was required in 690 infants (27.2%), a third dose was required in 216 infants (8.5%), and a fourth to sixth dose was required in 96 infants (3.8%); 9 infants (0.3%) required 7 to 14 doeses of surfactant.

### Association of LISA With Risks of Adverse Short-term Outcomes

Univariate comparison between infants receiving LISA and the subgroup not receiving LISA suggested lower rates of short-term complications in infants receiving LISA (Table 2). For example, pneumothorax occurred in 126 of 2534 infants receiving LISA (5.0%; 95% CI, 4.2%-5.9%) compared with 312 of 4008 patients not receiving LISA (7.8%; 95% CI, 7.0%-8.7%) (*P* < .001); the number of deaths from all causes among infants receiving LISA was 178 of 2534 (7.0%; 95% CI, 6.1%-8.1%) vs 433 of 4008 patients not receiving LISA (10.8%; 95% CI, 9.9%-11.8%) (*P* < .001). Rates of ICH were also higher among infants receiving LISA (723 of 2534 [28.5%; 95% CI, 26.8%-30.3%] vs 1450 of 4008 infants not receiving LISA (36.2%; 95% CI, 34.8%-37.8%) (*P* < .001) for any ICH.

**Table 2.** Short-term Outcomes Among Infants According to LISA Exposure^a^

Outcome	Infants receiving LISA (n = 2534)		Infants not receiving LISA					*P* value, LISA vs no LISA
	No. (%)	95% CI	No surfactant (n = 476)			ETT surfactant (n = 3532)		
			No. (%)	95% CI	No. (%)		95% CI	
Pneumothorax	126 (5.0)	4.2-5.9	6 (1.3)	1.0-2.5		306 (8.7)	8.1-9.8	<.001
Death in hospital, all cause	178 (7.0)	6.1-8.1	31 (6.5)	4.8-8.6		402 (11.4)	10.1-12.7	<.001
BPD	796 (31.4)	29.6-33.3	104 (21.8)	17.5-26.1		1578 (44.7)	43.2-46.2	<.001
BPD or death	944 (37.3)	35.4-39.2	130 (27.3)	23.6-31.1		1907 (54.0)	52.1-56.2	<.001
ICH^b^								
Any	723 (28.5)	26.8-30.3	75 (15.8)	13.0-18.7		1375 (39.0)	37.6-40.5	<.001
I	265 (10.5)	9.3-11.7	40 (8.5)	6.1-10.5		353 (10.0)	9.1-10.9	
II	180 (7.1)	6.2-8.2	19 (4.0)			387 (11.0)	10.0-11.9	
III	157 (6.2)	5.3-7.2	9 (1.9) (1.0-3.5)	2.5-6.0		295 (8.4)	7.8-9.1	
IV	121 (4.8)	4.0-5.7	7 (1.5)	1.0-2.5		339 (9.6)	8.7-10.5	
PVL	110 (4.4)	3.6-5.2	11 (2.3)	1.0-3.5		211 (6.0)	5.1-7.0	.03
Surgery for ROP	135 (5.5)	4.6-6.4	23 (4.9)	2.5-7.0		312 (9)	8.3-9.7	<.001

Abbreviations: BPD, bronchopulmonary dysplasia; ETT, endotracheal tube; ICH, intracerebral hemorrhage; LISA, less invasive surfactant administration; PVL, periventricular leukomalacia; ROP, retinopathy of prematurity.

^a^ Continuous variables were analyzed using the Mann-Whitney *U* test; dichotomous variables were analyzed using a 2-sided χ^2^ test. *P* values were derived for comparisons between the 2534 infants who received LISA and the 4008 infants not receiving LISA (the combination of those receiving no surfactant and those receiving surfactant through ETT).

^b^ Severity based on Papile grade, with I being the least and IV being the most severe.

**Figure 2.**
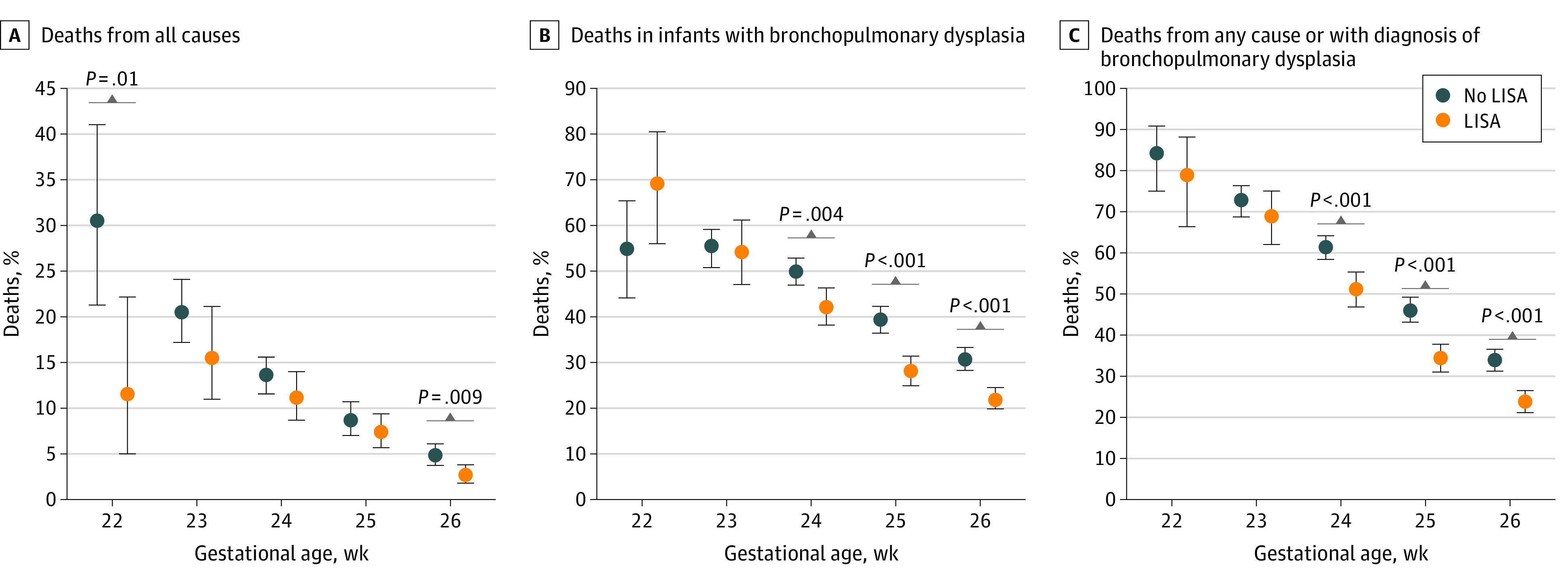
Percentages of German Neonatal Network–Enrolled Infants Not Surviving the Primary Stay in Hospital Values are presented as means and 95% CIs (whiskers). *P* values were derived using 2-sided χ^2^ tests. LISA indicates less invasive surfactant administration.

According to GA, use of LISA was associated with significantly lower mortality among infants born 22 weeks’ GA (6 [11.5%] of 52 infants receiving LISA vs 25 [30.5%] of 82 infants not receiving LISA; *P* = .01) and 26 weeks’ GA (27 [2.7%] of 1006 infants receiving LISA vs 59 [4.8%] of 1225 infants not receiving LISA; *P* = .009) (Figure 2A), reduced frequencies of BPD in infants 24 weeks’ GA (227 [42.2%] of 538 infants receiving LISA vs 554 [49.8%] of 1112 infants not receiving LISA; *P* = .004), 25 weeks’ GA (209 [28.1%] of 745 infants receiving LISA vs 417 [39.3%] of 1060 infants not receiving LISA; *P* < .001), and 26 weeks’ GA (220 [21.9%] of 1006 infants receiving LISA vs 376 [30.7%] of 1225 infants not receiving LISA; *P* < .001 (Figure 2B), and reduced rates of the composite outcome BPD or death in infants 24weeks’ GA (275 [51.1%] of 538 infants receiving LISA vs 682 [61.3%] of 1112 infants not receiving LISA; *P* < .001), 25 weeks’ GA (256 [34.4%] of 745 infants receiving LISA vs 488 [46.1%] of 1059 infants not receiving LISA; *P* < .001), and 26 weeks’ GA (239 [23.8%] of 1004 infants receiving LISA vs 415 [33.9%] of 1224 infants not receiving LISA; *P* < .001 (Figure 2C).

To address potential confounding factors of LISA as the primary exposure variable, we performed multivariate logistic regression including GA, SGA status, sex, multiple birth, inborn status (born at a tertiary care center), antenatal steroid use, and maximum Fio_2_ in the first 12 hours of life. In this model, LISA was associated with reduced risks of death from all causes during the primary stay in hospital (OR, 0.74; 95% CI, 0.61-0.90, *P* = .002), BPD (OR, 0.69; 95% CI, 0.62-0.78, *P* < .001), BPD or death (OR, 0.64; 95% CI, 0.57-0.72, *P* < .001), pneumothorax (OR, 0.66; 95% CI, 0.53-0.82, *P* < .001), ICH (OR, 0.78; 95% CI, 0.69-0.87, *P* < .001), and treatment requirement for retinopathy of prematurity (ROP; OR, 0.70; 95% CI, 0.57-0.87, *P* = .001) compared with infants without LISA) (Table 3).

**Table 3.** Multivariate Logistic Regression Models Showing Association of Outcomes With LISA After Adjustment for Possibly Confounding Variables

Outcome	Model 1^a^			Model 2^b^			Model 3^c^		
	No./total No.	OR (95% CI)	*P* value	No./total No.	OR (95% CI)	*P* value	No./total No.	OR (95% CI)	*P* value
All-cause in-hospital death	517/6214	0.74 (0.61-0.90)	.002	573/6169	0.74 (0.60-0.91)	.003	467/5229	0.93 (0.74-1.17)	.56
BPD	2362/6215	0.69 (0.62-0.78)	<.001	2342/6170	0.65 (0.57-0.73)	<.001	1930/5231	0.66 (0.58-0.76)	<.001
BPD or death	2840/6212	0.64 (0.57-0.72)	<.001	2817/6167	0.59 (0.53-0.67)	<.001	2318/5227	0.66 (0.58-0.75)	<.001
Pneumothorax	412/6207	0.66 (0.53-0.82)	<.001	412/6162	0.73 (0.58-0.92)	.008	327/5224	0.66 (0.51-0.88)	.002
Surgery for ROP	445/6070	0.70 (0.57-0.87)	.001	444/6025	0.78 (0.62-0.98)	.029	387/5105	0.69 (0.54-0.88)	.003
Any ICH	2057/6205	0.78 (0.69-0.87)	<.001	2045/6160	0.79 (0.70-0.89)	<.001	1708/5222	0.90 (0.79-1.03)	.12
PVL	316/6189	0.88 (0.69-1.12)	.29	316/6145	0.89 (0.69-1.15)	.38	277/5211	0.93 (0.71-1.22)	.60

Abbreviations: BPD, bronchopulmonary dysplasia; ICH, intracerebral hemorrhage; LISA, less invasive surfactant administration; OR, odds ratio; PVL, periventricular leukomalacia; ROP, retinopathy of prematurity.

^a^ Model 1: LISA as the primary exposure variable adjusted for gestational age, small-for-gestational-age status, sex, multiple birth, inborn status, antenatal steroid use, and maximum fraction of inspired oxygen in the first 12 hours of life.

^b^ Model 2: LISA as the primary exposure variable adjusted for the factors listed for model 1 plus maternal factors, including mode of delivery (elective cesarean delivery, vaginal birth, and emergency cesarean delivery), causes of preterm birth (labor, amniotic infection, preeclampsia/HELLP [highly elevated liver enzymes and low platelets], pathological Doppler result, and placental abruption), study site, and year of discharge.

^c^ Model 3: LISA as the primary exposure variable adjusted for all factors listed for model 2 plus surrogate markers of infant’s condition at birth (Apgar score at 5 minutes, arterial cord blood pH, and need for inotropes in the first 24 hours of life) plus participating center.

In a second model including 6170 infants, we added confounding variables that might affect the infant’s capacity for spontaneous breathing (eg, emergency cesarean delivery and causes of preterm delivery). We also considered the study site (tertiary care center, yes vs no) as well as year of discharge, which could be associated with LISA use. Our analysis found that LISA was associated with reduced risks of death from all causes (OR, 0.74; 95% CI, 0.60-0.91; *P* = .003), BPD (OR, 0.65; 95% CI, 0.57-0.73; *P* < .001), BPD or death (OR, 0.59; 95% CI, 0.53-0.67; *P* < .001), pneumothorax (OR, 0.73; 95% CI, 0.58-0.92; *P* = .008), surgery for ROP (OR, 0.78; 95% CI, 0.62-0.98; *P* = .029), and ICH (OR, 0.79; 95% CI, 0.70-0.89; *P* < .001) (Table 3).

In a third model, we additionally considered surrogate markers of early postnatal compromise (Apgar score at 5 minutes, arterial cord blood pH, and need for inotropes in the first 24 hours of life) in a subgroup of 5231 infants with full data sets. Our analysis found that LISA was associated with decreased risks of BPD (OR, 0.66; 95% CI, 0.57-0.76; *P* < .001), BPD or death (OR, 0.66; 95% CI, 0.58-0.75; *P* = .001), pneumothorax (OR, 0.66; 95% CI, 0.51-0.88; *P* = .002) and ROP (OR, 0.69; 95% CI, 0.54-0.88; *P* = .003), while no significant associations were noted for all-cause mortality or ICH (Table 3).

### Clinical Factors Associated With LISA Failure

We evaluated factors that were associated with LISA failure, defined as the need for IMV in the first 72 hours after initial CPAP support and LISA. We noted the following rates of LISA failure per week of GA: 22 weeks: 34 of 52 infants (65.4%; 95% CI, 51.9%-77.2%); 23 weeks: 132 of 193 infants (68.4%; 95% CI, 61.6%-74.6%); 24 weeks: 287 of 528 infants (53.3%; 95% CI, 49.1%-57.5%); 25 weeks: 344 of 745 infants (46.2%; 95% CI, 42.6%-49.8%); and 26 weeks: 380 of 1005 infants (37.8%; 95% CI, 34.9%-40.8%). In a multivariate logistic regression analysis including gestational age, sex, multiple birth, SGA status, antenatal steroid use, early-onset sepsis, Apgar score at 5 minutes, admission temperature, inborn status, study site, and highest Fio_2_ in the first 12 hours of life, the following factors were independently associated with reduced risk of LISA failure: each increasing week of gestation (OR, 0.79; 95% CI, 0.69-0.09; *P* < .001), female sex (OR, 0.71; 95% CI, 0.57-0.87; *P* = .001), antenatal steroid use (OR, 0.58; 95% CI, 0.36-0.95; *P* = .03), inborn status (OR, 0.38; 95% CI, 0.15-0.95; *P* = .04), and each increasing point of Apgar score at 5 minutes (OR, 0.79; 95% CI, 0.72-0.86; *P* < .001). In contrast, SGA status (OR, 1.64; 95% CI, 1.2-2.3; *P* = .006), study site (OR, 1.02; 95% CI, 1.01-1.02; *P* < .001), and maximum Fio_2_ in the first 12 hours of life (OR among those with maximum Fio_2_ of 0.30-0.59, 1.72; 95% CI, 1.35-2.2; *P* < .001, and OR among those with a maximum Fio_2_ of 0.60-1.0, 3.7; 0.60-1.0; 95% CI, 2.83-4.88; *P* < .001; reference, 0.21-0.29) were associated with increased risk of LISA failure, while admission temperature lower than 36.5 °C was not (OR, 1.25; 95% CI, 1-1.6; *P* = .05).

## Discussion

In this cohort study of the GNN including infants 22 weeks 0 days to 26 weeks 6 days’ GA, the use of LISA did not appear to pose a safety risk, as it was associated with lower risks of all-cause death, BPD, BPD or death, pneumothorax, surgery for ROP, and intracerebral hemorrhage. Factors associated with LISA failure, defined as need for IMV in the first 72 hours of life, included low GA, male sex, lack of antenatal steroid use, lower Apgar scores, SGA status, study site, and maximum Fio_2_ levels, which is in line with a previous study.^12^

The use of LISA was introduced in many GNN centers 10 to 15 years ago. This long-term experience and associated team confidence are important means for achieving broad, successful implementation of LISA within the primary context of stabilization.^13^ In this study, a large proportion of infants 22 to 26 weeks’ GA (38.7%) were treated with LISA. To our knowledge, no other large-scale comparative network data on LISA or MIST use in the most vulnerable preterm population have been reported to date. Given that 2-year follow-up data from the AMV (Avoid Mechanical Ventilation)^14^ and NINSAPP^15^ trials showed no adverse effects of LISA on neurodevelopment, LISA has replaced the use of the intubation, surfactant, and extubation protocol (known by the acronym INSURE) as the most frequent mode of surfactant application even in high-risk babies; INSURE involves the brief insertion of an endotracheal tube to administer surfactant to babies receiving CPAP, followed by a short period of positive pressure ventilation and extubation and a return CPAP.^16^ It typically requires analgesia or sedation, which may hamper the respiratory effort of preterm infants. As a consequence, extubation might be delayed in 10% to 17% of patients.^17,18^ Given the rapid physiological changes in the lung after surfactant administration, even a limited time of IMV may be harmful for the immature lung. The rationale behind the LISA concept includes several aspects: (1) prevention of lung injury by avoiding positive pressure ventilation; (2) reducing the risk of intubation trauma by using small-diameter catheters; (3) protecting the physiologic integrity of the larynx and glottis; and (4) supporting physiological adaptation, including lung recruitment, circulation, and regional tissue oxygenation.^7^ These ideas of supported spontaneous breathing and facilitated physiological transition have been shown to translate into improved clinical outcomes in RCTs^2,3^ and observational studies.^5,6^ The NINSAPP trial^3^ enrolled 211 eligible infants in Germany with GA criteria similar to those in our study. Inclusion criteria were spontaneous breathing and signs of respiratory distress (Fio_2_ >0.3 for saturation of peripheral oxygen, oxygen saturation >83%, and/or a Silverman score ≥5). Despite the differences in study size and design (RCT vs observational design and spontaneous breathing as an inclusion criterion in the NINSAPP trial), mortality rates were similar (approximately 9% vs 11% [LISA vs controls] in the NINSAPP trial and approximately 7% vs 11% [LISA vs no LISA] in our study).

In the first large-scale trial with blinded study design,^4^ OPTIMIST investigators demonstrated the benefits of MIST in terms of reduced risks of mechanical ventilation in the first 72 hours of life, pneumothorax, and BPD, although there were no increased risks of other important safety end points. The potential safety concern with regard to mortality in infants born at 25 to 26 weeks’ GA has not been confirmed by our observational data. There are differences in the contexts of the OPTIMIST trial^4^ and GNN studies.^1,3^ First, LISA is already part of the clinical routine in most neonatal intensive care units in German-speaking countries. Second, infants receiving LISA in our cohort had a 93.9% exposure to antenatal steroids and were delivered by elective cesarean section in 79.6% (Table 1). Third, early timing is a specific hallmark of the LISA concept (ie, it is already used in the delivery room in more than 80% of infants born at 22 to 26 weeks’ GA in the GNN). In the protocol developed at the University of Cologne,^9^ for example, infants are eligible for LISA if they are stable while receiving CPAP, have a heart rate greater than 120 beats per minute, and have oxygen saturation greater than 85%, regardless of Fio_2_ levels. Caution is needed in treating infants with early signs of cardiorespiratory compromise. In our multivariate logistic regression models including cord pH, Apgar score at 5 minutes, and the need for inotropes in the first 24 hours as markers of high risk at birth, we noted no significant associations of LISA use with mortality or ICH risk. However, the vast majority of extremely preterm babies are eligible for LISA, provided that the team can apply efficient noninvasive ventilation.^9^

In our experience, LISA is often performed early in the delivery room irrespective of Fio_2_ levels. So far, outcome data pertaining to this quasi-prophylactic approach are not yet available in a controlled setting. To overcome this research gap, the pro.LISA RCT has just started recruitment in Germany. This trial will assess whether, compared with selective surfactant treatment, prophylactic LISA use in preterm infants 25 to 28 weeks’ GA who are receiving noninvasive respiratory support is associated with better lung function at 5 years of age. In the context of the OPTIMIST trial, with neonatologists being blinded to whether infants received surfactant, further rescue treatment for increasing respiratory distress might be delayed. Under these circumstances, it is difficult to ascertain whether a potential clinical deterioration is a result of missing surfactant or missing effect of given surfactant. Fourth, surfactant administration is just one part of LISA, which comprises other components that may have differential effects and are difficult to account for in observational studies.^19,20,21,22^ For example, several GNN centers use caffeine in the delivery room and introduce colostrum feeding in the first hour of life, given the potential antioxidative effect.^9,23^

### Limitations

This study has limitations, including those inherent in an observational design and potential confounders that might have been missed in the applied regression models. Indication bias might have occurred, including unknown factors beyond the capacity for spontaneous breathing, availability of resources, or team preference. To address these concerns, our models included potential confounders that may affect spontaneous breathing, surrogate markers of infants’ compromise at birth, study site, and year of discharge to help explain variabilities in associations with LISA exposure and active perinatal care for infants younger than 24 weeks’ GA.^24^ Another limitation is selection bias, as nonenrolled but eligible infants had a higher likelihood of adverse outcomes, including death. Parents of nonenrolled infants were most often not approached for participation in the GNN, and it remains unknown whether their infants would have been stable enough for LISA.

It is possible that LISA may fail to avoid mechanical ventilation, and GA has been found to be associated with its success or failure.^12^ However, looking at favorable outcome data in the tiniest babies (eg, reduced risk of ICH), spontaneous breathing from birth might be of benefit, even if mechanical ventilation has to be started on day 2 or 3 of life. Other factors have also been found to contribute to LISA failure, including the absence of antenatal steroid use, high Fio_2_ thresholds, dose, timing of surfactant administration, low admission temperature, and early-onset sepsis, as well as practitioner and team experience.^12,13^ In the current study, SGA status appeared to be an additional risk factor for LISA failure. Consequently, improved risk stratification and targeted LISA approaches are required in the future, which underscores the importance of (1) refining the diagnosis of respiratory distress syndrome according to clinical, laboratory, and imaging evaluation; (2) determining the endogenous (genotypic and/or phenotypic) risk factors for respiratory distress syndrome in individual infants (eg, SGA status or early-onset sepsis); and (3) addressing modifiable environmental factors that contribute to the success of LISA (eg, antenatal steroid use, appropriate admission temperature,^12^ and efficient CPAP^25^).

### Conclusions

The results of this large-scale cohort study involving long-term LISA experience suggest that LISA is associated with reduced risks of adverse short-term outcomes in extremely preterm infants of less than 27 weeks’ GA and that mechanical ventilation in the first 72 hours may be prevented in more than half. There is still an urgent need to better define those babies at high risk for failing a treatment strategy that includes LISA. Randomized clinical trials are needed to assess the effects of prophylactic LISA in this vulnerable population.

## Notes

Supplement 1. **eTable 1.** Clinical Characteristics of Nonenrolled vs Enrolled Infants **eTable 2.** Outcomes of Nonenrolled vs Enrolled Infants per Gestational Week

Supplement 2. **Nonauthor Collaborators.** Members of the German Neonatal Network
