# Errors in a Figure

**DOI:** 10.1001/jamanetworkopen.2023.12619

**Published:** 2023-04-21

The Original Investigation “Association of Administration of Surfactant Using Less Invasive Methods With Outcomes in Extremely Preterm Infants Less Than 27 Weeks of Gestation,”^1^ published August 9, 2022, has been corrected to fix errors in Figure 2. The figure title now reads, “Percentages of Death, Bronchopulmonary Dysplasia (BPD), and the Combination of BPD or Death Among German Neonatal Network–Enrolled Infants,” and the figure panels have been amended to reflect these 3 outcomes. This article has been corrected.^1^

## References

[1] Härtel C, Herting E, Humberg, et al; German Neonatal Network. Association of administration of surfactant using less invasive methods with outcomes in extremely preterm infants less than 27 weeks of gestation. *JAMA Netw Open*. 2022;5(8):e2225810. doi:10.1001/jamanetworkopen.2022.25810 PMC9364126 35943742

