# Supplementary material for: Association of Administration of Surfactant Using Less Invasive Methods With Outcomes in Extremely Preterm Infants Less Than 27 Weeks of Gestation
Source: JAMA Netw Open. 2022 Aug 9;5(8):e2225810. doi: 10.1001/jamanetworkopen.2022.25810 (PMC9364126; doi:10.1001/jamanetworkopen.2022.25810)
Supplement: Supplement 1. — eTable 1. Clinical Characteristics of Nonenrolled vs Enrolled Infants eTable 2. Outcomes of Nonenrolled vs Enrolled Infants per Gestational Week [file jamanetwopen-e2225810-s001.pdf]

Supplemental Online Content

Härtel C, Herting E, Humberg A; German Neonatal Network. Association of administration of surfactant using less invasive methods with outcomes in extremely preterm infants less than 27 weeks of gestation. *JAMA Netw Open*. 2022;5(8):e2225810. doi:10.1001/jamanetworkopen.2022.25810

eTable 1. Clinical Characteristics of Nonenrolled vs Enrolled Infants

eTable 2. Outcomes of Nonenrolled vs Enrolled Infants per Gestational Week

This supplemental material has been provided by the authors to give readers additional information about their work.

© 2022 Härtel C et al. *JAMA Network Open.*

eTable 1. Clinical Characteristics of Nonenrolled Versus Enrolled Infants

**Gestational week**	**Non enrolled infants**	**Enrolled infants**	**p**
**Number of infants**	2832	6546	
**Gestational age, mean/SD, wk**	24.9/1.3	25.3/1.1	<0.001*
**Birth weight, mean/SD, g**	680/250	718/180	<0.001*
**Sex, female,** n, %	1242, 44.1	3035, 46.4	0.039
**Multiple birth, n, %**	707, 25.2	1989, 30.4	<0.001
**SGA, n, %**	597, 21.3	1113, 17.0	<0.001
**Inborn, n, %**	2674, 95.2	6098, 96.4	0.015
**BPD,** n; % (95%CI)	372, 13.5 (12-15)	2479, 37.9 (37-39)	<0.001
**BPD or death, n,** % (95%CI)	1754, 62.4 (61-64)	2984, 45.6 (44-47)	<0.001
**Death in hospital, all cause, n,** % (95%CI),	1428, 50.9 (49-53)	613, 9.4 (9-10)	<0.001
**ICH, n,** % (95%CI)	1038, 39.7 (38-42)	2173, 33.3 (32-34)	<0.001

Non-enrolled: 2821/2832 baseline datasets were available for all items except BPD (2764) and ICH (2617); enrolled: 6545/6546 full datasets available except for ICH (6258). P-values were derived from chi-square test

© 2022 Härtel C et al. *JAMA Network Open.*

eTable 2. Outcomes of Nonenrolled Versus Enrolled Infants per Gestational Week

**Gestational week**	**22 weeks**	**23 weeks**	**24 weeks***	**25 weeks**	**26 weeks**
**Number of infants** Non-enrolled/enrolled	196/134	496/720	718/1652	693/1806	718/2233
**BPD,** n; % (95%CI) Non-enrolled/enrolled, p-value	9; 4.4 (2-8) / 81; 60.4 (52-69) <0.001	60, 12.4 (10-16) 394, 54.8 (51-58) <0.001	113, 16.1 (14-19) 781, 47.3 (45-50) <0.001	100, 14.7 (12-18) 627, 34.7 (33-37) <0.001	91, 12.7 (11-15) 596, 26.7 (25-29) <0.001
**BPD or death, n,** % (95%CI) Non-enrolled/enrolled	186; 94.9 (91-97) 110; 82.1 (75-88) <0.001	427, 86.4 (83-89) 516, 71.7 (68-75) <0.001	474, 66.1 (63-70) 959, 58.1 (56-60) <0.001	383, 55.7 (52-59) 745, 41.3 (39-44) <0.001	284, 39.7 (36-43) 654, 29.3 (28-31) <0.001
**Death in hospital, all cause, n,** % (95%CI), Non-enrolled/enrolled	179; 91.3 (87-95) 31; 23.1 (17-31) <0.001	379, 76.7 (73-80) 138, 19.2 (16-22) <0.001	380, 53.1 (49-57) 212, 12.8 (11.3-14.5) <0.001	290, 42.2 (39-46) 146, 8.1 (7-9) <0.001	200, 28.0 (25-31) 86, 3.9 (3-5) <0.001
**ICH, n,** % (95%CI) Non-enrolled/enrolled	5; 40.8 (32-50) 73; 54.2 (46-63) 0.034	219, 50.8 (46-56) 338, 47.1 (43-51) 0.2	308, 45.0 (41-49) 635, 38.6 (36-41) 0.004	268, 39.8 (36-44) 562, 31.2 (29-32) <0.001	194, 27.4 (24-31) 567, 25.4 (24-27) 0.28

ICH database for non-enrolled infants: 22 wk: 12, 23 wk: 431, 24 wk: 685, 25 wk: 674, 26 wk: 707.

p-values were derived from Chi-square test as comparison of enrolled vs. non-enrolled infants

© 2022 Härtel C et al. *JAMA Network Open.*
